# The preclinical and phase 1 development of the novel oral cathepsin C inhibitor BI 1291583

**DOI:** 10.1183/23120541.00725-2023

**Published:** 2024-03-25

**Authors:** James D. Chalmers, Philipp Badorrek, Claudia Diefenbach, Harald Kögler, Wiebke Sauter, Stefan Kreideweiss, Jens M. Hohlfeld

**Affiliations:** 1 Division of Molecular and Clinical Medicine, University of Dundee, Dundee, UK; 2 Department of Clinical Airway Research, Fraunhofer Institute for Toxicology and Experimental Medicine (ITEM), Hannover, Germany; 3 Boehringer Ingelheim Pharma GmbH & Co. KG, Biberach, Germany; 4 Boehringer Ingelheim International GmbH, Ingelheim, Germany; 5 Department of Respiratory Medicine, Hannover Medical School, Hannover, Germany; 6 German Centre for Lung Research (DZL), Hannover, Germany

## Abstract

Bronchiectasis is a heterogeneous lung disease characterised by chronic inflammation, infection, impaired mucociliary clearance and progressive structural lung damage [1, 2]. These features interact in a “vicious vortex”, leading to exacerbations and declining pulmonary function with associated morbidity and mortality [1, 2].

## Shareable abstract

**Preclinical and phase 1 study results indicate that BI 1291583 is a reversible, highly potent and highly selective CatC inhibitor that markedly inhibits active NSP production in a dose-dependent manner, supporting phase 2 trials in bronchiectasis patients** https://bit.ly/47PZ8E5

*To the Editor:*

Bronchiectasis is a heterogeneous lung disease characterised by chronic inflammation, infection, impaired mucociliary clearance and progressive structural lung damage [1, 2]. These features interact in a “vicious vortex”, leading to exacerbations and declining pulmonary function with associated morbidity and mortality [1, 2].

Neutrophilic inflammation and an imbalance between neutrophil-derived serine proteases (NSPs; neutrophil elastase (NE), proteinase 3 (PR3) and cathepsin G) and their anti-proteases are central features of bronchiectasis [1, 3]. High, uncontrolled NSP levels, which have been shown to impair mucociliary clearance, drive mucus hypersecretion, cause structural lung damage and weaken host defences, thereby contributing to each component of the vicious vortex [3]. Effective blockade of NSPs could therefore ameliorate each of these components.

As cathepsin C (CatC; also known as dipeptidyl peptidase 1) activates NSPs in the bone marrow during myelopoiesis [4], CatC inhibition is expected to reduce NSP activity in the lungs of patients with bronchiectasis, thereby restoring the protease/antiprotease balance. No drug is currently licensed for the treatment of bronchiectasis; therefore, there is a high unmet need for a novel treatment that reduces inflammation and improves symptoms and quality of life. BI 1291583, a novel CatC inhibitor, is currently being investigated in a phase 2 trial in adults with bronchiectasis (Airleaf) [5, 6]. Here, we summarise the preclinical and phase 1 development of BI 1291583.

Preclinical investigations involved *in vitro* assessment of the binding and inhibition of CatC, and assessment of NSP inhibition. We assessed the binding kinetics of BI 1291583 to isolated human CatC using surface plasmon resonance (pH 4.5). To determine the specificity of BI 1291583 for CatC, we measured the inhibition of cathepsins C, B, F, H, K, L and S by conversion of fluorescent substrates. Additionally, we assessed the specificity of BI 1291583 against 33 unrelated proteases from four classes using enzyme assays, and validated fluorometric or photometric techniques. To determine whether inhibition of CatC leads to inhibition of active NSP production, we measured NE activity by the conversion of a fluorescent substrate in lysates of the human myeloid progenitor cell line U937 (which produces high levels of activated NE) exposed to increasing concentrations of BI 1291583. Finally, we assessed the *in vivo* inhibition of active NE and PR3 production by exposing mice to 0.00005–5 mg·kg^−1^ BI 1291583 once daily over 11 days, followed by bacterial lipopolysaccharide challenge. NSP activity was determined in bronchoalveolar lavage fluid (BALF) neutrophils by conversion of fluorescent substrates. Exposure to BI 1291583 in the target bone marrow compartment and plasma of the mouse model was measured *via* liquid chromatography-tandem mass spectrometry.

BI 1291583 bound human CatC in a covalent, reversible manner, with a mean dissociation constant of 0.43 nM and a mean half-life of 5.19 min. CatC enzymatic activity was inhibited with a half-maximal inhibitory concentration (IC_50_) of 0.9 nM and with high selectivity (>6000-fold selectivity *versus* related cathepsins, and no relevant inhibition or stimulation of unrelated proteases). Production of active NE in U937 cells was inhibited in a concentration-dependent manner with an IC_50_ of 0.7 nM whilst maintaining a cell viability of 93–98%. In mice BALF neutrophils, production of active NE (figure 1a) and PR3 was almost completely inhibited in a dose-dependent manner (99% and 94% in the 5 mg·kg^−1^ dose group, respectively). BI 1291583 distributed preferentially to the bone marrow, with a bone marrow-to-plasma exposure ratio of up to 100 at efficacious doses. The physicochemical properties of BI 1291583 (balanced basicity and partition coefficient) in combination with the strong binding affinity of BI 1291583 with CatC (dissociation constant (Kd) of 0.43nM) are believed to have led to the high bone marrow-to-plasma distribution at efficacious doses.

**FIGURE 1:**
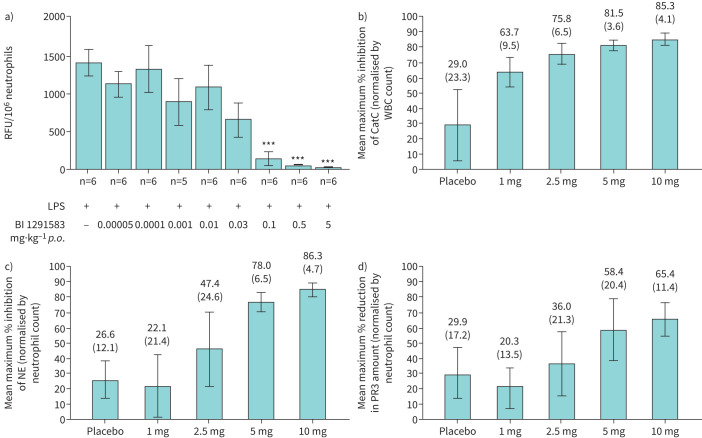
Summary of key preclinical and phase 1 data. a) Effect of treatment with BI 1291583 on NE activity in mouse BALF neutrophil lysate. b) Mean maximum percentage inhibition of CatC after multiple oral administrations of BI 1291583 in healthy volunteers. c) Mean maximum percentage inhibition of zymosan-stimulated neutrophil elastase activity after multiple oral administrations of BI 1291583. d) Mean maximum percentage reduction in PR3 amount after multiple oral administrations of BI 1291583. ***: p<0.001 compared with vehicle. In a, error bars indicate standard error of the mean. In b–d, error bars indicate standard deviation. BALF: bronchoalveolar lavage fluid; CatC: cathepsin C; LPS: lipopolysaccharide; NE: neutrophil elastase; *p.o.*: by mouth; PR3: proteinase 3; RFU: relative fluorescence units; WBC: white blood count.

Together, these results demonstrate that BI 1291583 is a highly potent inhibitor of CatC, leading to relevant inhibition of NSPs and supporting further investigation. As such, we carried out five phase 1 trials of BI 1291583 in healthy Caucasian adults: three single-blind, partially randomised, placebo-controlled, parallel-group trials, one of which investigated single rising doses (SRD) (1–40 mg; n=54 (NCT03414008) [7]) and two of which investigated once-daily multiple rising doses (MRD) (1 mg, 2.5 mg; n=24 (NCT03868540) [8]; 5 mg, 10 mg; n=24 (NCT04866160) [9]); an open-label, randomised, single-dose, two-period, two-sequence crossover bioavailability study investigating subjects under fed and fasted conditions (7.5 mg; n=12 (NCT03837964) [10]); and an open-label, 2.5 mg single-dose, two-period, fixed-sequence crossover drug–drug interaction study with and without itraconazole (n=14 (NCT03890887) [11]). As BI 1291583 is almost exclusively metabolised by cytochrome P450 3A4 (CYP3A4) and is a substrate of a P-glycoprotein (P-gp), inhibitors of these enzymes could result in clinically relevant increases in BI 1291583 exposure. As such, itraconazole, a strong CYP3A4 and P-gp inhibitor, was used in the drug–drug interaction study. The doses selected in the SRD study covered the assumed sub-therapeutic and therapeutic ranges, and included a safety margin; the doses selected in the MRD studies covered the assumed sub-therapeutic, therapeutic and supra-therapeutic ranges, and included a safety margin; the dose selected for the food effect study was the assumed therapeutic dose at the time of study conduct; and a dose of 2.5 mg BI 1291583 was selected in the drug–drug interaction study in order to ensure that BI 1291583 plasma concentrations, even in the case of substantial increase when co-administered with itraconazole, were within the range of concentrations that were explored in the SRD trial and that had been associated with good safety and tolerability. Sample size was not based on a power calculation for any of the five phase I trials; the same sizes selected were, in general, considered sufficient for the exploratory analyses performed, including the evaluation of safety and pharmacokinetics. Primary end-points were investigator-judged drug-related adverse events (AEs) for the SRD and MRD studies, and BI 1291583 exposure for the food effect and drug–drug interaction studies. AEs of special interest (AESIs) included hyperkeratosis and periodontal disease; patients with Papillon–Lefèvre syndrome, a rare genetic disease caused by loss-of-function mutations in the CatC gene, often present with hyperkeratosis and severe periodontal disease [12], and these events should be monitored in trials of CatC inhibitors. However, complete loss of CatC enzymatic activity is not expected with BI 1291583. Secondary end-points for all trials included pharmacokinetic parameters, as well as CatC and NE activity and PR3 amount in blood for the SRD and MRD studies specifically.

Across the trials, almost all the AEs reported were mild or moderate in intensity, with no reported serious AEs, AESIs or deaths. Two severe treatment-emergent AEs were reported (one gastrointestinal infection event (SRD study) and one joint injury event (food effect study)), and one patient was prematurely withdrawn due to AEs (C-reactive protein increased and thrombophlebitis), but all events were considered unrelated to BI 1291583. Similar rates of skin exfoliation, considered drug related by the investigator, were observed in the BI 1291583 *versus* placebo groups (MRD study 1: two out of 18 *versus* one out of 6; MRD study 2: one out of 18 *versus* 0 out of six). No infections or changes in white blood cell counts were reported as being related to treatment. BI 1291583 was readily absorbed, and exposures increased supraproportionally over the dose range investigated (MRD studies), with an apparent terminal half-life of 60.2 h (40 mg dose). Exposure at 5 mg exceeded that predicted by preclinical studies to be required for 99% inhibition of CatC. BI 1291583 exposure was generally similar under fed and fasted conditions; however, co-administration of BI 1291583 with multiple doses of itraconazole resulted in an approximate twofold increase in exposure. BI 1291583 inhibition of CatC was dose-dependent, with a maximum inhibition of 96% in the SRD 30 mg dose group and 85% in the MRD 10 mg dose group (figure 1b). Subsequent inhibition of NE activity was observed, with a maximum inhibition of 86% (10 mg dose; MRD study) (figure 1c). Additionally, decreased levels of PR3 were observed, with a maximum decrease of 65% (10 mg dose; MRD study) (figure 1d).

In summary, preclinical studies demonstrate that BI 1291583 is a highly potent inhibitor of CatC that leads to marked inhibition of NE and PR3. Furthermore, it distributes to the target bone marrow compartment at an exposure up to 100× higher than in plasma at efficacious doses. Across the five phase 1 trials in healthy subjects, BI 1291583 was found to be safe, well tolerated and readily absorbed and to exhibit dose-dependent inhibition of CatC, thereby inhibiting downstream NE activity and decreasing PR3 levels.

To date, only two other CatC inhibitors have reached clinical trials in patients. HSK31858 is being assessed in a phase 2 trial in patients with non-cystic fibrosis bronchiectasis (NCT05601778), and the oral, reversible inhibitor brensocatib (formerly AZD7986/INS1007) is being assessed in a phase 3 trial for patients with bronchiectasis (NCT04594369). In light of the published preclinical data for brensocatib [13], our data suggest that BI 1291583 may also be a promising candidate for bronchiectasis treatment. In the phase 1 study of brensocatib [14], 25 mg once-daily administration resulted in a 49% decrease in NE activity in blood; in the phase 2 study, this dose resulted in ∼90% inhibition of sputum NE activity [15] and in a subsequent exploratory analysis, a 53% inhibition of sputum PR3 activity was reported [16]. A recent pharmacokinetic/pharmacodynamic analysis of brensocatib [17] showed that patients achieving an NE level below the limit of quantification (BLQ) had a greatly reduced risk of exacerbation. However, even at the 25 mg once-daily dose, many patients did not achieve an NE level BLQ. The high bone marrow:plasma distribution we have demonstrated in the mouse model may help to achieve greater proportions of patients with substantially suppressed airway NSPs. Indeed, in the phase 1 trials, we observed a marked decrease in blood NE activity with a relatively low (5 mg) dose of BI 1291583; therefore, this dose may result in high NE inhibition in sputum in phase 2 studies. A 5 mg dose of BI 1291583 also resulted in a 58% decrease in blood PR3 levels. This broad inhibition of NSPs may add further beneficial therapeutic potential in patients with bronchiectasis.

Marked skin desquamation events were partly responsible for the termination of a previous CatC inhibitor [18], and drug-related skin events are reported in phase 1 and 2 trials of brensocatib [14, 15]. Skin events occur independently of the action of CatC in the bone marrow, and likely occur as a result of the role that CatC plays in the processing of keratins in keratinocytes, and thus in maintaining the structural integrity of plantar and palmar epidermal surfaces [19], rather than as a result of NSP inhibition. The lack of drug-related skin events during our phase 1 trials, potentially due to high bone marrow:plasma distribution, reinforces the potential of BI 1291583.

In conclusion, BI 1291583 is a reversible, highly potent and highly selective inhibitor of CatC that markedly inhibits production of active NSPs in a dose-dependent manner. It is safe, well tolerated and, crucially, did not increase the incidence of drug-related skin exfoliation. A multinational phase 2 trial of BI 1291583 in adults with bronchiectasis is ongoing (Airleaf) [5].

## References

[1] Flume PA, Chalmers JD, Olivier KN. Advances in bronchiectasis: endotyping, genetics, microbiome, and disease heterogeneity. Lancet 2018; 392: 880–890. doi:10.1016/S0140-6736(18)31767-7 30215383 PMC6173801

[2] McShane PJ, Naureckas ET, Tino G, et al. Non-cystic fibrosis bronchiectasis. Am J Respir Crit Care Med 2013; 188: 647–656. doi:10.1164/rccm.201303-0411CI 23898922

[3] Oriano M, Amati F, Gramegna A, et al. Protease-antiprotease imbalance in bronchiectasis. Int J Mol Sci 2021; 22: 5996. doi:10.3390/ijms22115996 34206113 PMC8199509

[4] Adkison AM, Raptis SZ, Kelley DG, et al. Dipeptidyl peptidase I activates neutrophil-derived serine proteases and regulates the development of acute experimental arthritis. J Clin Invest 2002; 109: 363–371. doi:10.1172/JCI0213462 11827996 PMC150852

[5] ClinicalTrials.gov. A study to test whether different doses of BI 1291583 help people with bronchiectasis. 2022. Date last updated: 25 January 2023. Date last accessed: 5 July 2023. https://clinicaltrials.gov/ct2/show/NCT05238675

[6] Chalmers JD, Gupta A, Chotirmall SH, et al. A Phase 2 randomised study to establish efficacy, safety and dosing of a novel oral cathepsin C inhibitor, BI 1291583, in adults with bronchiectasis (Airleaf™). ERJ Open Res 2023; 9: 00633-02022. 37465817 10.1183/23120541.00633-2022 PMC10351677

[7] ClinicalTrials.gov. This study tests how different doses of BI 1291583 are taken up in the body and how well they are tolerated. 2018. Date last updated: 11 October 2020. Date last accessed: 2 February 2023. https://clinicaltrials.gov/ct2/show/NCT03414008?term=NCT03414008&draw=2&rank=1

[8] ClinicalTrials.gov. A study in healthy men and women to find out how well different doses of BI 1291583 are tolerated. 2019. Date last updated: 23 November 2021. Date last accessed: 2 February 2023. https://clinicaltrials.gov/ct2/show/NCT03868540?term=NCT03868540&draw=2&rank=1

[9] ClinicalTrials.gov. A trial in healthy men and women to find out how well different doses of BI 1291583 are tolerated. 2021. Date last updated: 7 December 2021. Date last accessed: 2 February 2023. https://clinicaltrials.gov/study/NCT04866160

[10] ClinicalTrials.gov. A study to test how food influences the amount of BI 1291583 in the blood of healthy men. 2019. Date last updated: 5 November 2023. Date last accessed: 2 February 2023. https://clinicaltrials.gov/ct2/show/NCT03837964?term=NCT03837964&draw=2&rank=1

[11] ClinicalTrials.gov. A study in healthy men to test how itraconazole influences the amount of BI 1291583 in the blood. 2019. Date last updated: 11 November 2023. Date last accessed: 2 February 2023. https://clinicaltrials.gov/ct2/show/NCT03890887?term=NCT03890887&draw=2&rank=1

[12] Dhanrajani PJ. Papillon-Lefevre syndrome: clinical presentation and a brief review. Oral Surg Oral Med Oral Pathol Oral Radiol Endod 2009; 108: e1–e7. doi:10.1016/j.tripleo.2009.03.016 19540439

[13] Doyle K, Lonn H, Kack H, et al. Discovery of second generation reversible covalent DPP1 inhibitors leading to an oxazepane amidoacetonitrile based clinical candidate (AZD7986). J Med Chem 2016; 59: 9457–9472. doi:10.1021/acs.jmedchem.6b01127 27690432

[14] Palmer R, Maenpaa J, Jauhiainen A, et al. Dipeptidyl peptidase 1 inhibitor AZD7986 induces a sustained, exposure-dependent reduction in neutrophil elastase activity in healthy subjects. Clin Pharmacol Ther 2018; 104: 1155–1164. doi:10.1002/cpt.1053 29484635 PMC6282495

[15] Chalmers JD, Haworth CS, Metersky ML, et al. Phase 2 trial of the DPP-1 inhibitor brensocatib in bronchiectasis. N Engl J Med 2020; 383: 2127–2137. doi:10.1056/NEJMoa2021713 32897034

[16] Cipolla D, Zhang J, Korkmaz B, et al. Dipeptidyl peptidase-1 inhibition with brensocatib reduces the activity of all major neutrophil serine proteases in patients with bronchiectasis: results from the WILLOW trial. Respir Res 2023; 24: 133. doi:10.1186/s12931-023-02444-z 37198686 PMC10189992

[17] Chalmers JD, Usansky H, Rubino CM, et al. Pharmacokinetic/pharmacodynamic evaluation of the dipeptidyl peptidase 1 inhibitor brensocatib for non-cystic fibrosis bronchiectasis. Clin Pharmacokinet 2022; 61: 1457–1469. doi:10.1007/s40262-022-01147-w 35976570 PMC9553789

[18] Miller BE, Mayer RJ, Goyal N, et al. Epithelial desquamation observed in a phase I study of an oral cathepsin C inhibitor (GSK2793660). Br J Clin Pharmacol 2017; 83: 2813–2820. doi:10.1111/bcp.13398 28800383 PMC5698569

[19] Nuckolls GH, Slavkin HC. Paths of glorious proteases. Nat Genet 1999; 23: 378–380. doi:10.1038/70472 10581013

